# Supplementary material for: Eudraguard® Natural and Protect: New “Food Grade” Matrices for the Delivery of an Extract from Sorbus domestica L. Leaves Active on the α-Glucosidase Enzyme
Source: Pharmaceutics. 2023 Jan 16;15(1):295. doi: 10.3390/pharmaceutics15010295 (PMC9861284; doi:10.3390/pharmaceutics15010295)
Supplement: Supplementary file 1 [file pharmaceutics-15-00295-s001.zip › pharmaceutics-2014348-supplementary.pdf]

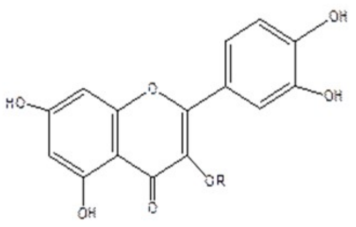

	R
**1**	Glc
**2**	Glc(1→2)Rha
**3**	Rha(1→6)Glc
**4**	Xyl(1→2Rha)
**5**	Rha
**6**	Gal

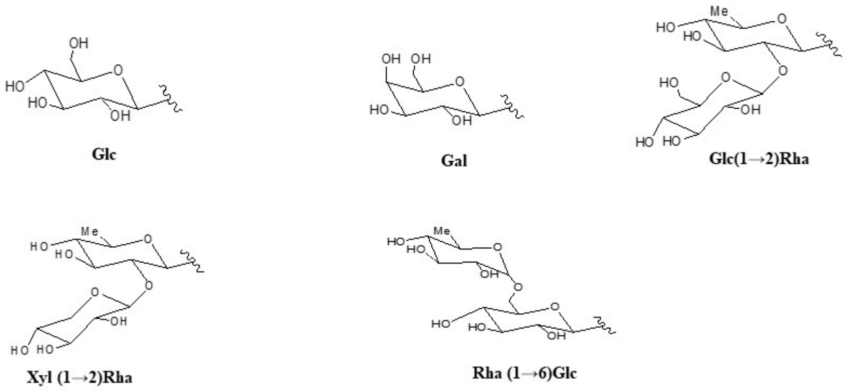

Figure S1. Structure of compounds isolated from SOE extract

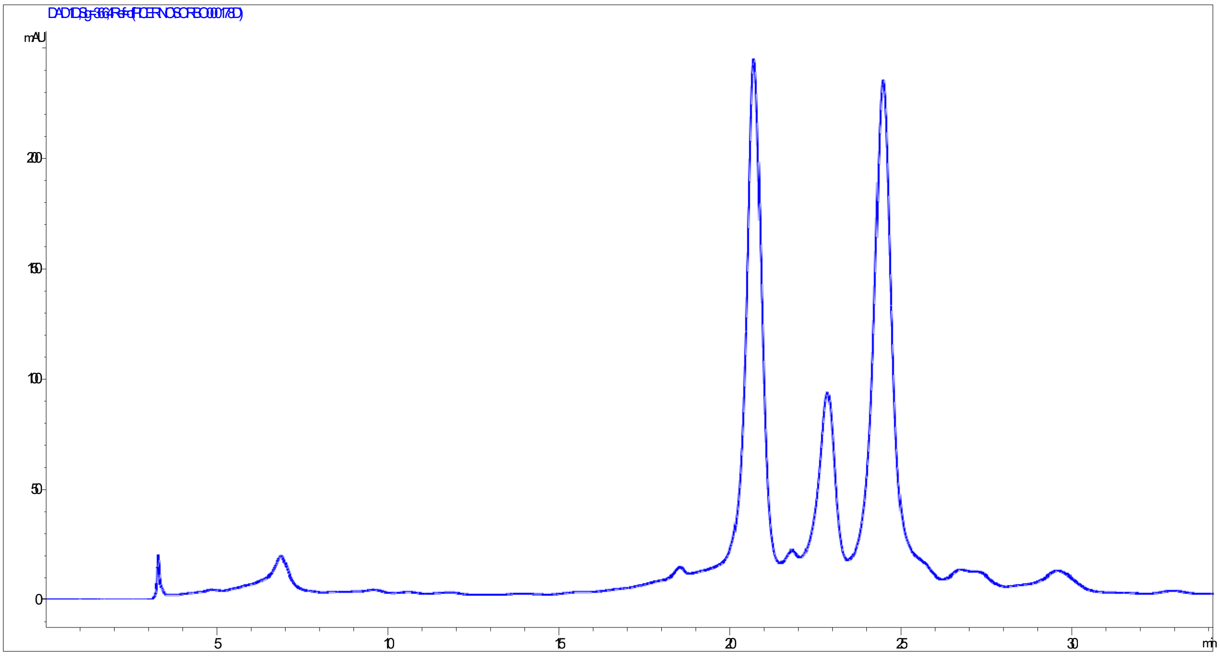

Figure S2. HPLC-UVDAD profile of SOE (366 nm)

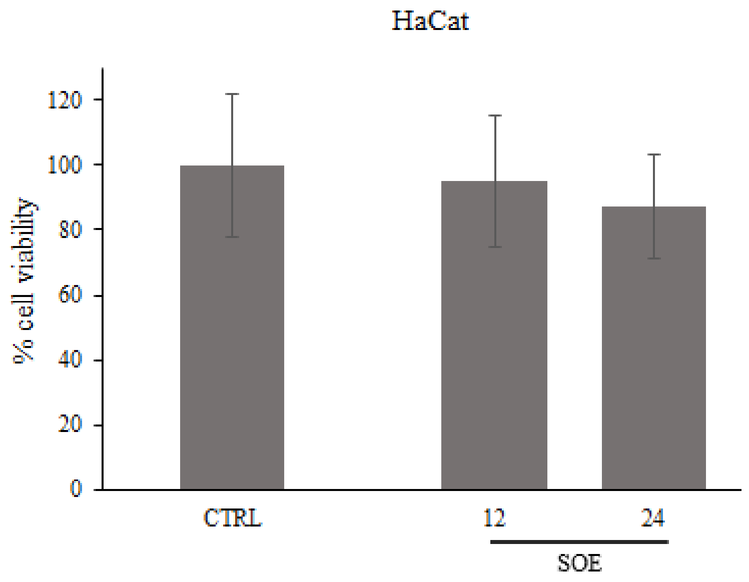

Figure S3. Viability of HaCaT cells (Immortalized human normal keratinocytes) after 48 h of incubation with different concentrations of SOE (12 or 24 mg/mL), determined by MTT assay. The data show the mean±S.D. of three independent experiments performed in triplicate and are expressed as % of control (% of Ctr, cells untreated). Statistical analysis was made using ANOVA followed by the Bonferroni test, a p value < 0.05 was considered statistically significant.
